# Developing protein arginine methyltransferase 1 (PRMT1) inhibitor TC-E-5003 as an antitumor drug using INEI drug delivery systems

**DOI:** 10.1080/10717544.2020.1745327

**Published:** 2020-03-26

**Authors:** Pengcheng Zhang, He Tao, Liyang Yu, Lixiao Zhou, Chenggang Zhu

**Affiliations:** a College of Life Sciences, Zhejiang University, Hangzhou, China;; b Institute of Hygiene, Zhejiang Academy of Medical Science, China

**Keywords:** NBCA, INEI, TC-E-5003, new anti-tumor drugs, intratumoral chemotherapy

## Abstract

Injectable implants with the ability to form in situ are one of the most promising carriers for the delivery of chemotherapeutic drugs to tumor sites. We have reported a novel injectable in situ-forming implant system composed of n-butyl-2-cyanoacrylate (NBCA), ethyl oleate, along with the sol-gel phase transition. The chemotherapeutic drug-loaded injectable NBCA ethyl oleate implant (INEI) exhibited excellent therapeutic efficacy for local chemotherapy. Herein, we utilize this INEI to carry N, N′-(Sulfonyldi-4,1-phenylene)bis(2-chloroacetamide) (TE-C-5003), which is a selective protein arginine methyltransferase 1 (PRMT1) inhibitor, to treat the lung cancer mice model. The *in vitro* experiment shows that TE-C-5003 has a good anti-tumor effect on lung cancer (IC_50_ = 0.7022 µM for A549; IC_50_ = 0.6844 µM for NCL-H1299) and breast cancer (IC_50_ = 0.4128 µM for MCF-7; IC_50_ = 0.5965 µM for MDA-MB-231). Anti-tumor experiments in animal models showed that the average growth inhibition rate of xenografted human lung cancer cells by the TE-C-5003-loaded INEI (40% NBCA) was 68.23%, which is far more than TC-E-5003 alone (31.76%). Our study further confirms that INEI is an effective technique to improve the anti-tumor effect. The druggability of small molecule compounds can be improved with the help of the mentioned technology. Also, TC-E-5003 may be developed as a broad spectrum anti-tumor drug.

## Introduction

Cancer is a serious disease worldwide. According to GLOBOCAN data, there were18.1 million new cancer cases (17.0 million excluding nonmelanoma skin cancer) and 9.6 million cancer deaths (9.5 million excluding non-melanoma skin cancer) in 2018. Lung cancer is the commonly diagnosed cancer (11.6% of the total cases) and the leading cause of cancer death (18.4% of the total cancer deaths) (Bray et al., 2018). Non-small-cell lung cancer (NSCLC) is the predominant subgroup of lung cancer with a high opportunity for recurrence and metastasis (IARC, 2018). In addition to surgery, therapy includes adjuvant chemotherapy, which is considered standard for cancer and still under debate for stage II disease (Matsuda et al., 2018). However, several anti-cancer drugs have rapid plasma clearance resulting in minimal tumor exposure (Brudno & Mooney, 2015). New drug delivery approaches are effective in order to provide a high local concentration of the anticancer drug, enhance the efficacy of chemotherapy, as well as to reduce systemic side effects. Numerous papers have been published in the field of drug delivery carriers that validate their superior anti-tumor effect (Chakravarty et al., 2015; Croft et al., 2018; Giordano et al., 2017; Lu et al., 2014; Raza et al., 2018; Werner et al., 2013). Recently, we reported on a novel biodegradable N-Butyl-2-cyanoacrylate-based injectable in situ-forming implants, formed from n-butyl-2-cyanoacrylate (NBCA) and ethyl oleate. The novel injectable in situ-forming implant system (INEI) can be triggered to solidify by body fluid and blood. It shows an excellent sustained-release effect with both hydrophobic and hydrophilic drugs. We have shown that the sol-gel phase transition, the relevant irregular particles polymerized by the NBCA, the inner hole of the implant, and the hardness of the implant pore changes of the implant as the concentration of NBCA increased (Wu et al., 2017).

Also, we can control the rate of degradation of the implant by adjusting the ratio of the NBCA in the system, to release the chemotherapy drug for a long time at a tumor site, reduce systemic side effects and the paclitaxel-loaded INEI or epirubicin-loaded INEI showed effectual anti-tumor symptoms. Some papers have reported that NBCA is biodegradable, biocompatible, nontoxic, and used in some clinical applications. (Jang et al., 2017; Kong et al., 2018; Sugawara et al., 2019).

N,N′-(Sulfonyldi-4,1-phenylene)bis(2-chloroacetamide) (TC-E-5003) is a selective protein arginine methyltransferase 1 (PRMT1) inhibitor (IC_50_ =1.5 *μ*M). It does not display activity against CARM1 and SET7/9 methyltransferases, and also inhibits the growth of MCF7 breast cancer cells and LNCaP prostate cancer cells. It also blocks androgen-induced gene expression in LNCaP cells (Bissinger et al., 2011; Heinke et al., 2008). Nine PRMT members have been identified that exert arginine methylase activity in human cells (Yang & Bedford, 2013). The family members are classified into three types: type I, type II, and type III. Type I includes PRMT1, −2, −3,−4,−6, and −8; type II includes PRMT5 and PRMT9 whereas type III includes only PRMT7 (Branscombe et al., 2001). Most of the arginine methylation is catalyzed by PRMT1 (mainly asymmetric methyl) and PRMT5 (mainly symmetric methyl). There are differences in expression levels and regulatory mechanisms in cancer cells. PRMT1 can be detected in breast cancer, lung cancer, colon cancer, bladder cancer, and acute myeloid leukemia, but its expression may be disordered in different types of cancer (Cheung et al., 2007; Feng et al., 2013; Goulet et al., 2007; Mathioudaki et al., 2008). Our previous study demonstrated that paclitaxel-loaded INEI and epirubicin-loaded INEI had enhanced the inhibitory effect on the tumor and reduced the systemic side effects more than free paclitaxel and epirubicin in tumor xenografts. In this paper, TC-E-5003 was not used in clinical procedures to treat tumor that was loaded in the INEI system to treat cancer. TC-E-5003-loaded-INEI could inhibit the proliferation of A549 cells *in vitro*. It is worth noting that TC-E-5003-loaded-INEI obtained excellent anti-tumor efficacy and reduced side system effect in comparison with the free TC-E-5003, indicating that the INEI system holds great potential in the development of new anti-tumor drugs.

## Materials and methods

### Materials

**Figure 1.**
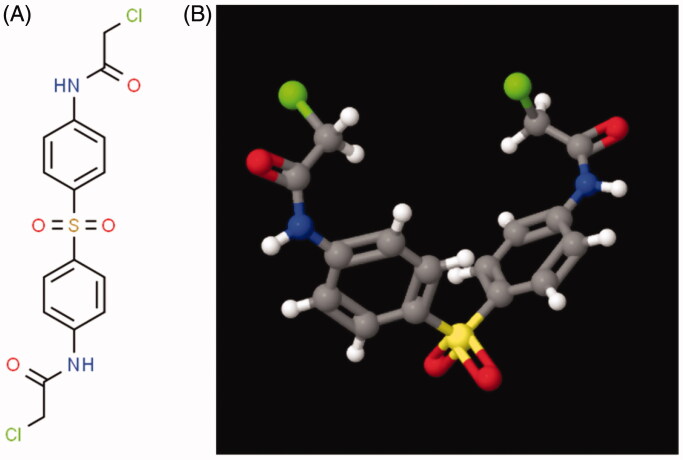
N,N′-(Sulfonyldi-4,1-phenylene)bis(2-chloroacetamide) (TC-E-5003) structural formula. (A) 2D structural formula of N,N′-(Sulfonyldi-4,1-phenylene)bis(2-chloroacetamide) (TC-E-5003). (B) N,N′-(Sulfonyldi-4,1-phenylene)bis(2-chloroacetamide) (TC-E-5003) of 3 D.

N-Butyl-2-cyanoacrylate was purchased from Jinpeng Chemical Co., LTD, Zhejiang, China. Ethyl oleate was purchased from Aladdin Chemicals Co., LTD, Shanghai, China. N,N′-(Sulfonyldi-4,1-phenylene)bis(2-chloroacetamide) (TE-C-5003, Figure 1) was purchased from Active Motif co., LTD, Shanghai China. Epirubicin and paclitaxel were purchased from Haizheng Pharmaceutical Co., LTD, Zhejiang, China. Human breast cancer cell lines (MCF7, MDA-MB-231) and lung cancer cell lines (A549, NCL-H1299) were obtained from the cell bank of the Chinese Academy of Sciences, Shanghai, China. Female nude mice (4 weeks old) and ICR mice were purchased from Vital River Laboratory Animal Technology Co., Ltd. Beijing, China and maintained under SPF conditions for 1 week before the study in Zhejiang University Animal Experimental Center. DMSO and Acetonitrile were purchased from Sigma Aldrich. The Annexin V-FITC Apoptosis Detection Kit and The Cell Counting Kit-8 were purchased from Beyotime Co., LTD, Shanghai China. All of the other reagents and solvents were purchased from Sinopharm Chemical Reagent Co., LTD., Mainland, China, and used as received. All the studies comply with the principles of care and use of laboratory animals from the Institutional Animal Care and Use Committee of Zhejiang University Health Science Center.

### Preparation of the TC-E-5003-INEI

TC-E-5003 was dissolved in DMSO to a final concentration of 50 mg/mL and the volume of DMSO is 0.2 mL. After it was added to a solution of ethyl oleate with a volume of 0.36 mL. The solution was removed from the resulting mixture using a freeze-drying system for 6 h. Then, the TE-C-5003-ethyl oleate suspension was sonicated three times using a probe-type sonicator at 200 W for 5 min in an ice bath. The pulse was turned off for 10 s every working 4 s to avoid high temperature. Finally, the volume of TC-E-5003-ethyl oleate was kept 0.36 mL and was stored at 4 °C for subsequent use. NBCA (12 uL) was added to the plate bottom tube with 18 µL TC-E-5003-ethyl oleate suspension, and the NBCA concentrate was 40%. Then, 3 uL saline was added and left to sit at room temperature for 24 h.

### *In vitro* drug release

One hundred microliters of INEI that contained either 40% NBCA and 0.5 mg TC-E-5003 or 40% NBCA and 0.1 mg epirubicin were added to a pre-swollen dialysis tube. Then, the dialysis tubes were immersed in a glass bottle with a PBS solution (V = 50 mL) and TE-C-5003 powder or 0.1 mg epirubicin were added to the corresponding dialysis tubes as a control. The glass bottles were placed in a thermostatic shaker (the speed at 40 rpm/min, 37 °C). At each time point (12 h, 1 d, 2 d, 3 d … 10d), the solution around the dialysis tube was collected and replaced by an equal volume of the same medium. The collected solution was freeze-dried and redissolved with the same DMSO. The concentration of TC-E-5003 dissolved in DMSO was assayed by HPLC-UV using a C18 column with the following mobile phase: methanol–acetonitrile-water (4:3:3, v/v) at a flow rate of 1 mL/min and a detection wavelength of 228 nm, with a sample injection volume of 20 mL.

### Cell line and cell culture

Human breast cancer cell lines (MCF7, MDA-MB-231) and lung cancer cell lines (A549, NCL-H1299) were obtained from the cell bank of the Chinese Academy of Sciences, Shanghai, China, and their identity was verified before experiments. Cells were cultured in DMEM containing 10% FBS (Gibco) and 1% penicillin/streptomycin and incubated at 37 °C in a humidified atmosphere with 5% CO_2_. Cell lines were free of mycoplasma contamination.

### *In vitro* cytotoxicity assay

The cells were seeded at 1 × 104 cells per well in 200 μL DMEM complete medium on 96-well plates. Cells were treated with various concentrations (0–10 uM) of TC-E-5003. DMSO was used as the negative control. Cytotoxic effects were analyzed at 48 h. The half-maximal inhibitory concentration (IC_50_) of each extract was derived by a nonlinear regression model (curve-fit) based on the sigmoidal dose-response curve (variable slope) and computed using GraphPad Prism 7.

The Annexin V-FITC Apoptosis Detection Kit was used to determine cell apoptosis and cell necrosis. Briefly, the cells were seeded at 1 × 104 cells per well on 96-well plates containing 200 μL complete medium and allowed to attach for 24 h. After adding 0.6 µM of TC-E-5003 and TC-R-5003-INEI, the viability of the cells were measured after 3 h, 48 h and 96 h. After that, cells were double-stained with two probes (Annexin-V-FITC and PI), enabling the simultaneous determination of apoptosis and necrosis in a sample. Fluorescence intensity was measured on a fluorescence microscope and analyzed by fluorescence-activated cell sorting (FACS).

### *In vivo* anti-tumor activity

All animals were maintained in a specific pathogen-free (SPF) facility, with Nude/ICR mice at an age of approximately 6 weeks (∼20 g body weight) used. Thirty-five ICR mice were used to exam the toxic of TC-E-5003 and TC-E-5003-INEI. Five mice were incorporated into each group. Each group of mice was given an injection of 30 uL of either saline, Ethyl oleate (subcutaneous injection), TC-E-5003 (0.5 mg, 1.0 mg, 2.0 mg) and Ethyl oleate (subcutaneous injection), TC-E-5003 (1.0 mg, 2.0 mg) and Ethyl oleate (subcutaneous injection). The weight of each mice was measured every 2 days. Terminal bleeds were taken via cardiac punctures on day 25. Serum levels of blood, white boot and the residue of TC-E-5003.

A549 cell xenografts were subcutaneously generated at the hind flank upon anesthesia mediated by isoflurane inhalation. When the tumors grew to approximately 100 mm^3^, thirty-five nude mice were randomly divided into five groups, with seven mice in each group. After the mice had been weighed and their tumors were measured, each group of mice was given a single antitumor injection of 30 µL of either saline, INEI with 40%NBCA, INEI with 0.5 mg TC-E-5003 and 0.15 mg epirubicin-40% NBCA of INEI. The treatment efficacy was assessed by measuring the volume of the tumor with a caliper every 2 days. The living state, body weight, and tumor volume (V = (length)×(width)^2^/2, where length (mm) and width (mm) are the tumor dimensions at the longest point and the widest point) of the mice in each group were documented every two day (Wu et al., 2017). On day 28, all mice peripheral blood was gathered via cardiac puncture. Blood was transferred into a 1.5 mL Eppendorf tube, followed by centrifugation at 12000 rpm for 10 min at 4 °C. Clear supernatants containing serum were collected and transferred into a sterile 1.5 mL Eppendorf tube, save in −80 °C. After then, all of the mice were sacrificed, and all of the tumors were weighed and ground up. The drug residue was then extracted and assayed by HPLC. All animal experiments were performed in compliance with the Zhejiang University Guide for the Care and Use of Laboratory Animals.

### Histological examination

To further evaluate the anti-tumor efficacy of TC-E-5003-INEI, the tumors were dissected from the mice and some of the tumors were sectioned for an H&E assay for the pathology (Madan et al., 2019; Warembourg & Leroy, 2000; Yamashita & Okada, 2005).

### INEI-paclitaxel toxicity test* in vivo*

Thirty-two healthy SD rats, half male and half female, were divided into control group, intravenous paclitaxel injection group, INEI low-dose group and INEI high-dose group according to gender and weight, with 8 animals in each group half (Extended Data Table 1). Animals in the intravenous paclitaxel injection group were given a 2 mg/mL paclitaxel injection in the tail vein at a dose of 10 mg/kg with a dose volume of 10 mL/kg in a single dose; the INEI-paclitaxel group was given 2 mg/mL subcutaneously The paclitaxel sustained-release implants were dosed at 10 mg/kg and 20 mg/kg, respectively, and the administered volumes were 5 mL/kg (INEI low-dose group) and 10 mL/kg (INEI high-dose group). During the test, the clinical symptoms of the animals were observed daily, body weight was measured on Days 1, 3, 5, 7 and the animals were dissected for clinical pathology (hematology, serum biochemistry) on Day 8 (Extended Data Table 2), gross anatomical examinations and weighing of major organs Calculate the visceral coefficient.

### Statistical data analysis

Statistical analysis was conducted with Prism 8.0 (GraphPad). All *in vitro* experiments were performed in triplicates and triplicate samples were analyzed in each experiment, and animal studies were performed with 7 mice per group. Unless otherwise indicated, data in the figures are presented as mean ± SD, and statistical significance was determined by unpaired two-tailed Student’s *t*-test. Two-tailed unpaired Student’s *t*-tests with 95% confidence intervals were used to calculate all *p* values. Significance was set at *p* < 0.05.

## Results

### Preparation of the TC-E-5003–ethyl oleate suspension and NBCA implant preparation

Preparation of the TC-E-5003–ethyl oleate suspension and NBCA implant preparation N,N′-(Sulfonyldi-4,1-phenylene)bis(2-chloroacetamide) (TC-E-5003) is a selective protein arginine methyltransferase 1 (PRMT1) inhibitor (IC50 = 1.5 μM), and it has not been applied to tumor therapy alone, although it has the anticancer efficacy for a wide spectrum of cancer (MCF-7 breast cancer and LNCaP prostate cancer cells). However, its anticancer unique mechanism is not clear, so we chose it as the new anticancer drug to exam our INEI system. TC-E-5003 is a hydrophobic molecule and has poor solubility in water. To ensure that paclitaxel does not block the injection needle, the TC-E-5003 used in implants must be composed of tiny particles.

**Figure 2.**
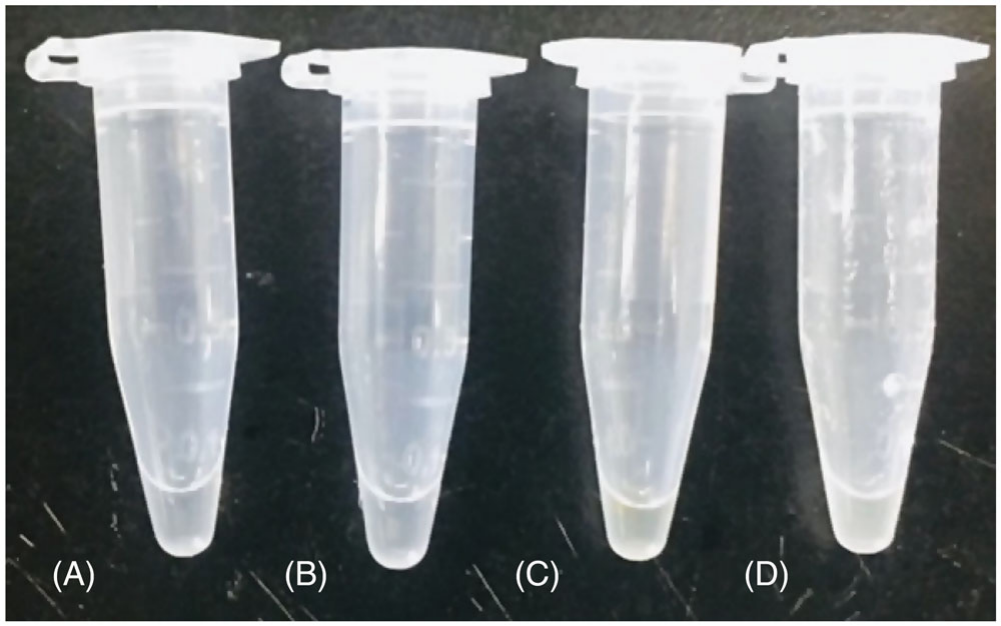
(A) 200 µL NBCA. (B) 200 µL ethyl oleate. (C) 100 µL 0.5 mg TC-E-5003 (DMSO)- ethyl oleate. (D) 100 µL 40%INEI (0.5 mg TC-E-5003).

To prepare the TC-E-5003 suspension, a DMSO solution of TC-E-5003 was added to ethyl oleate, and then the DMSO was removed by freeze-drying. After ultrasonication, the paclitaxel-ethyl oleate suspension was slightly clearer (Figure 2). The size and the shape of the paclitaxel particles in the suspension did not change after storage at 4 °C for 2 months. The irregular particles polymerized by the NBCA became smaller, the inner hole of the implant also became smaller, and the hardness of the implant increased with the increases in NBCA concentration. (Wu et al., 2017). We choose 40% NBCA to make the TC-E-5003-INEI *in vitro*.

### Effect of treatment with TC-E-5003 and TC-E-5003-INEI on cancer cells viability

**Figure 3.**
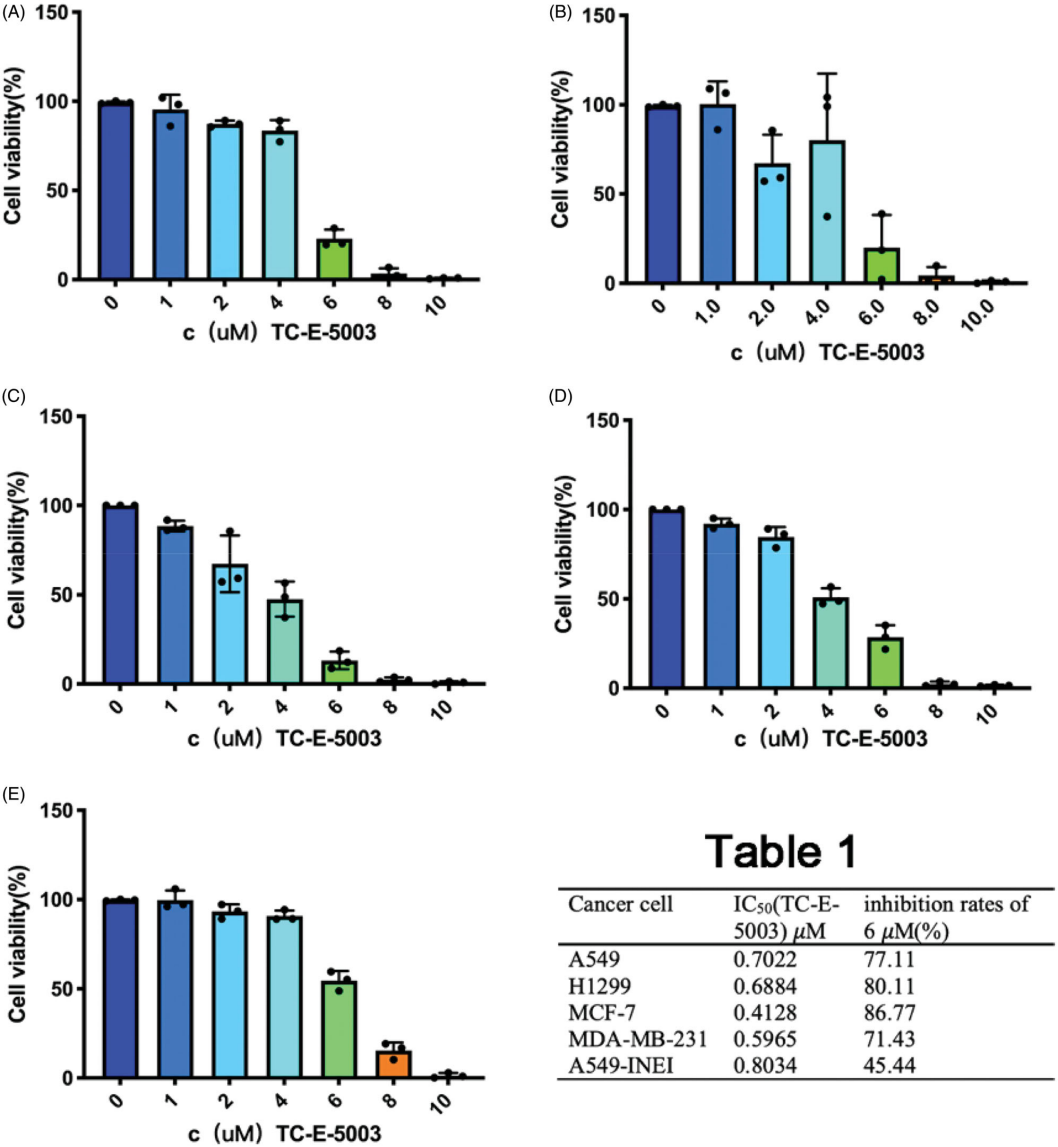
Graphical representation of cell viability data. (A-D) Cell viability changes of A549, H1299, MCF-7 and MDA-MB-231 cells after adding different concentrations of TC-E-5003. (E) Cell viability changes of A549 cells after adding different concentrations of TC-E-5003 in INEI system.

**Figure 4.**
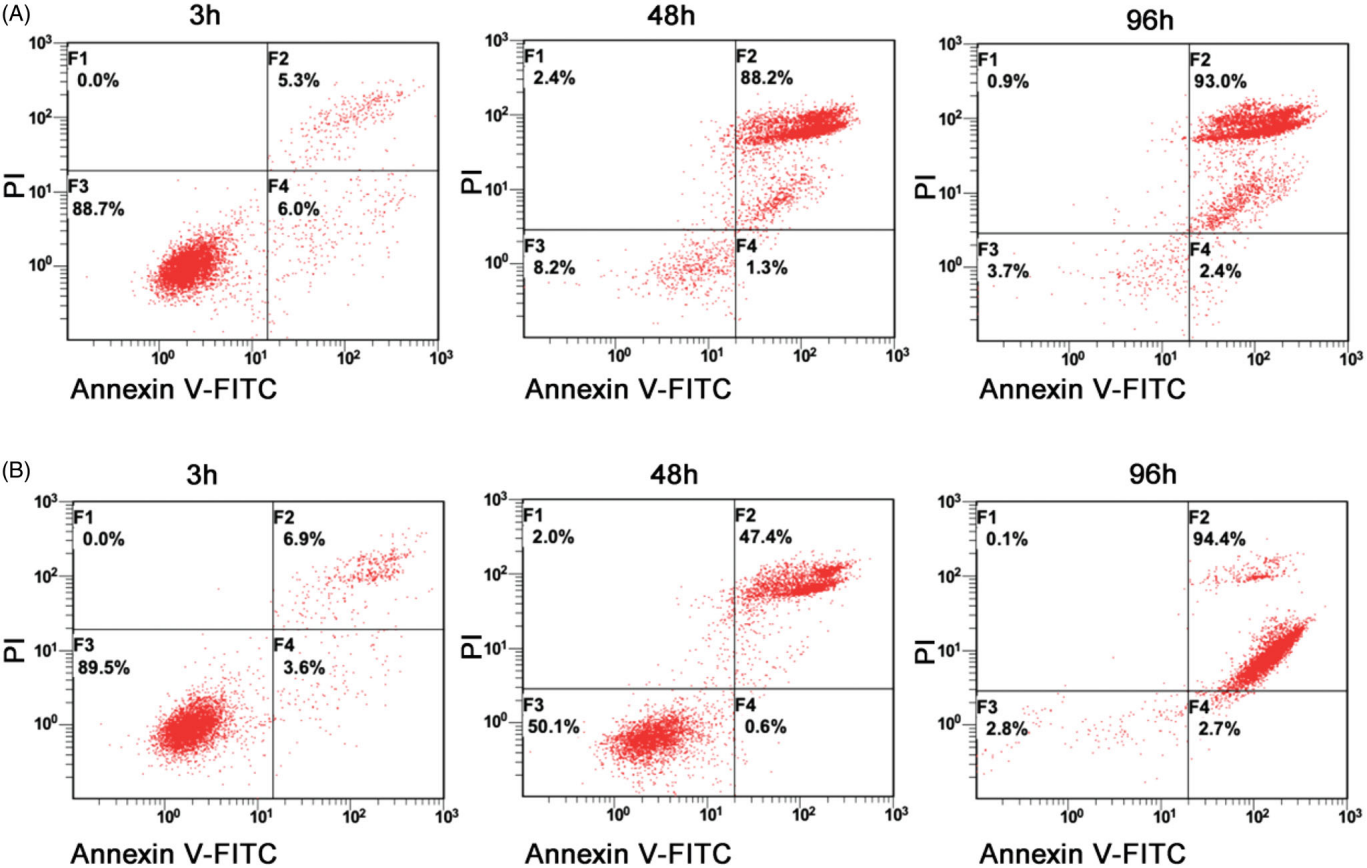
TC-E-5003 and TC-E-5003-INEI showed stronger activity to induce A549 cells apoptosis and cell necrosis. (A) A549 cells were stimulated with TC-E5003 (0.6 µM) for 3 h, 48 h and 96 h, then cells were stained using propidium iodide (PI) and annexin V and analyzed by fluorescence-activated cell sorting (FACS). (B) A549 cells were stimulated withINEI-TC-E-5003 (0.6µM) for 3 h, 48 h and 96 h, then cells were stained using propidium iodide (PI) and annexin V and analyzed by fluorescence-activated cell sorting (FACS).

To further confirm the cell proliferation inhibition effect of TC-E-5003 and TC-E-5003-INEI, we conducted the corresponding assay using the CCK-8 method. The inhibition of TC-E-5003 on A549, H1299, MCF-7 and MDA-MB-231 cells was significant. When TC-E-5003 concentration was at 6.0 *μ*M, the inhibition rates for A549, A549-INEI, H1299, MCF-7, and MDA-MB-231 (77.11%, 45.44%, 80.11%, 86.77%, 71.43%) were significantly stronger than that in 4 *μ*M (the inhibition rates:16.47%, 9.14%, 19.81%, 52.43%, 49.09%). However, treatment with TC-E-5003-INEI was not very high cell viability inhibition as compared with that of the TC-E-5003 for A549 cells. After 48 hours later, a part of TC-E-5003 was freed from INEI. The IC_50_ of TC-E-5003 for A549, H1299, MCF-7and MDA-MB-231 were 0.7022 *μ*M, 0.6884 *μ*M, 0.4128 *μ*M, and 0.5965 *μ*M. The IC_50_ of TC-E-5003-INEI for A549 was 0.8034 *μ*M. Data were statistically analyzed and graphically represented in Figure 3(A–E). Herein, flow cytometry was used to test whether TC-E-5003 and TC-E-5003-INEI showed stronger activity to induce A549 cells apoptosis and cell necrosis. As shown in Figure 4, TC-E-5003 induce apoptosis of A549 cells and resulted in significantly higher cell necrosis rates (89.5%) in comparison with the cells treated with TC-E-5003-INEI or INEI (48%) after 48 h. The result showed that no significant changes in the cellular viability of A549 cell exposed to TC-E-5003 and TC-E-5003-INEI were observed after 3 h and 96 h. These results indicated that INEI could not enhance the anticancer activity of TC-E-5003 to induce A549 cells necrosis in a short time. We think that the drug has not played a killing effect in a short time. The results showed that TC-E-5003 had a good inhibition on the proliferation of cancer cells, and TC-E-5003-INEI did not increase toxicity for A549 cell. The INEI system can slow the release rate of the drug.

### *In vitro* drug release

**Figure 5.**
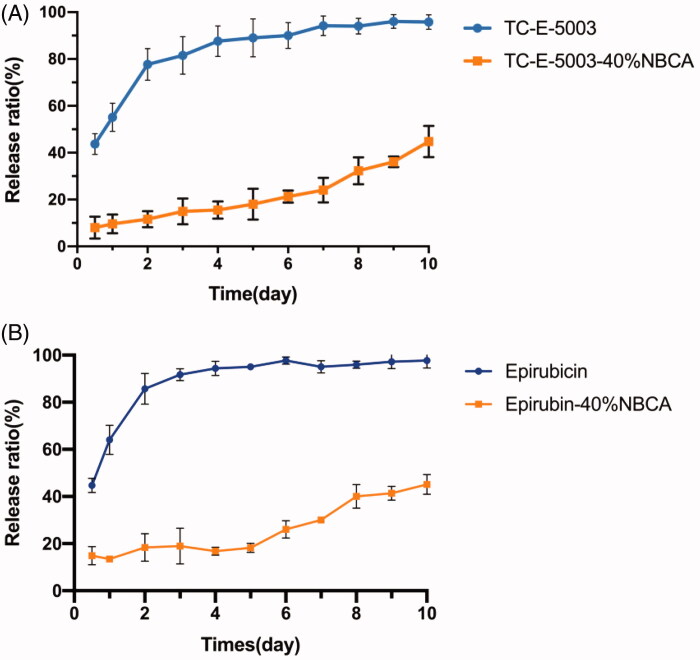
The release ratio of TC-E-5003 and epirubin loaded in an INEI with 40% NBCA. (A) Release ratio of TC-E-5003 and TC-E-5003 loaded in an INEI with 40% NBCA; (B) release ratio of epirubicin and epirubicin loaded in an INEI with 40% NBCA. The concentration of released TC-E-5003 in the Phosphate buffered saline was quantified as described in the Materials and methods section. The amount of initially incorporated TC-E-5003 (0.5 mg) is defined as 100%. Each data point represents the mean ± SD of triplicate measurements.

To evaluate the sustained release of TC-E-5003 from the INEI system, the drug release experiments have been performed *in vitro*. To increase the solubility of TC-E-5003 in solution and accelerate the experimental process, we performed an *in vitro* release test in 1 mol/L pH 7.4 Phosphate buffered saline. Figure 5(A) shows the release profiles of TC-E-5003 from the 40% INEI system and TC-E-5003 only. The results demonstrated that the initial burst effect was lower (8.1% versus 43.7%) for the TC-E-5003-INEI versus TC-E-5003. After 6d, the total TC-E-5003 released from the 40% INEI group was approximately 21.3%, whereas the total TC-E-5003 released from the TC-E-5003 free group was 90%, which was close to a complete release of the drug. Besides, the total amount of TC-E-5003 released from the 40% INEI group by the 10th day was 44.8%. Figure 5(B) shows the release profiles of epirubicin form the 40% INEI system and epirubicin only. The total amount of epirubicin released from the 40% INEI group by the 10th day was 46.9%. These results suggest the INEI system has an obvious function in slowing drug release.

### *In vivo* therapeutic efficacy of TC-E-5003-INEI

**Figure 6.**
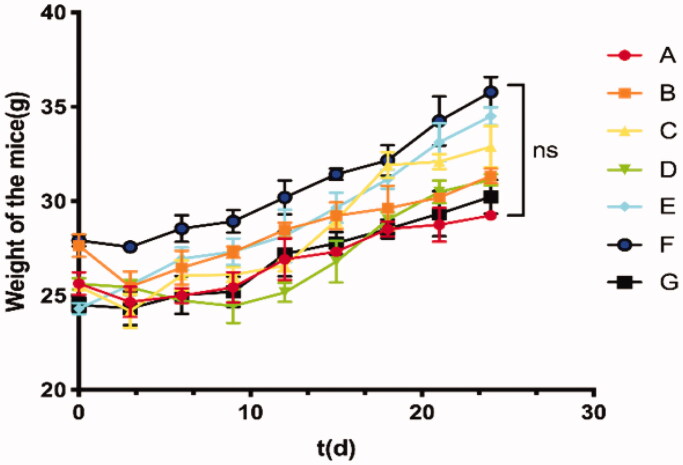
Toxic side effects of TC-E-500 on IRC mice. (A) 30 µL of either saline (subcutaneous injection). (B) 30 µL Ethyl oleate (subcutaneous injection). (C–E) TC-E-5003 (0.5 mg, 1.0 mg, 2.0 mg) and Ethyl oleate (subcutaneous injection); (F-G) TC-E-5003 (1.0 mg, 2.0 mg) and Ethyl oleate (subcutaneous injection).

**Figure 7.**
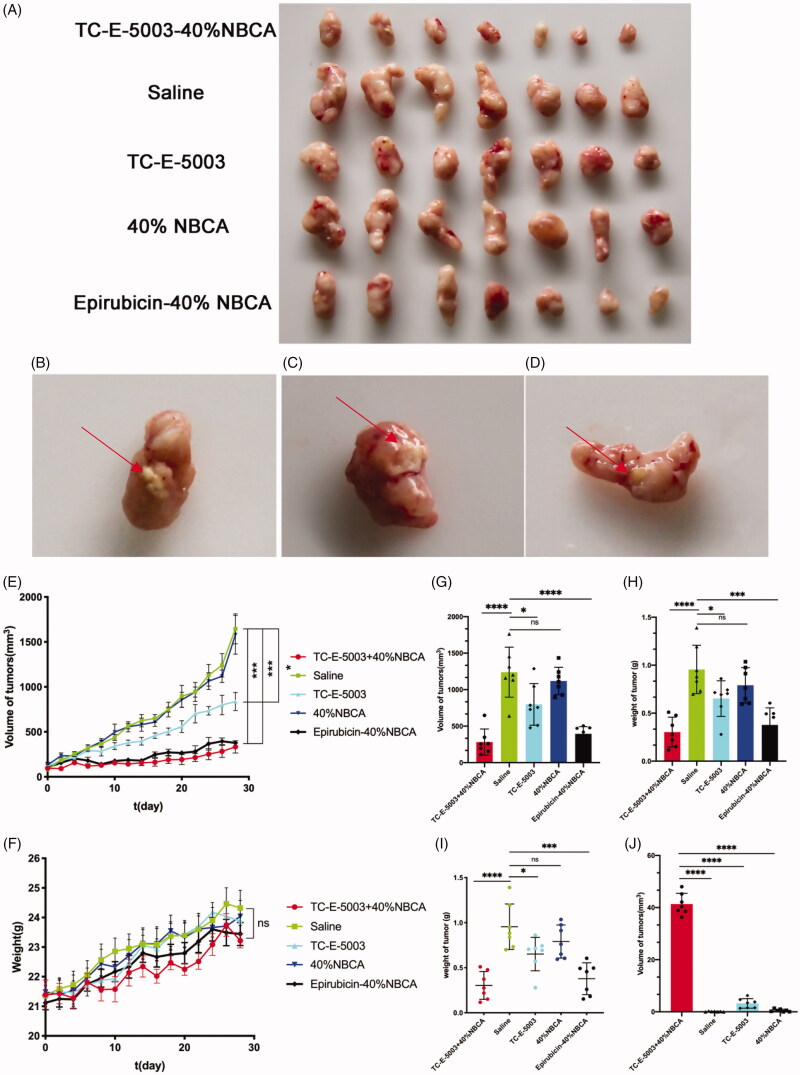
The INEI system improves the chemotherapy efficiency of TC-E-5003. (A–D) The photographs of tumors in each group and partially residual INEI system. The tumors were peeled off 24 h after the final dose. (E) Tumor volume curve in the groups that the mice were treated with different preparations. The tumor volume was documented every other day during the treatment. Volumes of tumors were measured with a caliper every two days. The volume of the tumor was calculated by the formula: V = (length) × (width)^2^/2, (L (mm) and W (mm) are the tumor dimensions at the longest point and widest point). (F) Bodyweight curve in the groups that the mice were treated with different preparations. The body weight was documented every other day during the treatment. After 28 days, all the mice were sacrificed, and the tumors from each group were weighed (G-I). The weight and volume of tumor masses upon INEI treatment. Mice were subjected to a single inti-tumoral injection of 30 µL of saline (green), 40% NBCA of INEI (blue), TC-E-5003 (containing 0.5 mg, sky blue), 0.5 mg TC-E-5003-40% NBCA of INEI (red) and 0.15 mg epirubicin-40% NBCA of INEI (black). (J) The residual of TC-E-5003 in tumor masses. *p < .05, ****p < .0001.

In this study, we established the ICR mouse model to investigate the toxic effect of TC-E-5003. The results show that low doses and high doses of TC-E-5003 did not influence the weight of the mouse. No mouse was found deceased (Figure 6). A549 tumor xenograft was used to assess the anti-tumor efficacy of TC-E-5003 and TC-E-INEI. Mice were assigned to five groups randomly. When tumors reached 100 mm^3^ in volume, mice were treated with a series of preparations including Saline, 40%NBCA, TC-E-5003, TC-E-5003-40%NBCA and epirubicin-40%NBCA. This was applied on A549 tumor-bearing mice at the TC-E-5003 and epirubicin dose of 0.5 mg and 0.15 mg. Bodyweight and tumor volume were recorded every two days during the treatment according to the tumor volume curves shown in Figure 7. After twenty-eighth day of treatment, the saline group show the average tumor volume at around 895 mm^3^ and the average weight of the tumors was 0.955 g representing the natural growth speed of the tumor. In the 40%NBCA groups, the average tumor volume was around 865 mm^3^ and the average weight of the tumors was 0.791 g. In the free TE-C-5003 group, the TC-E-5003 loaded 40% NBCA implant group and epirubicin loaded 40% NBCA implant group, the average volume of the tumors was 557 mm^3^, 192 mm^3^, and 225 mm^3^; the average weight of the tumor was 0.652 g, 0.378 g and 0.303 g. The tumor inhibition efficiency of each group was shown in Figure 7. These animal model experiments show that NBCA implants alone do not inhibit the growth of the tumor. The TC-E-5003 have an antitumor effect (31.76%), while the effect of tumor inhibition was 68.23% when TC-E-5003 loaded in 40%NBCA. These results showed that the INEI is efficient at enhancing tumor inhibition compared with free TC-E-5003. The drugs may be released into the tumors more slowly, thereby increasing their drug exposure time. Compared with the direct inti-tumoral injection of the drug solution, the drugs released from the INEI had better tumor therapeutic effects. These animal experiments show that the INEI system alone does not alter the natural progression of the tumor, whereas the TC-E-5003-INEI and epirubicin-INEI have a significant anti-tumor effect. The result can vividly show that INEI not only can enhance the anti-tumor effect of conventional chemotherapy drugs but also can use to develop new anti-tumor drugs. The INEI system has a significant advantage that drugs can be released into the tumors more slowly, thereby increasing their drug exposure time. Systematic toxicity was evaluated by measuring body weight changes over time during the treatment period Figure 7(F), The mice body weight in all the groups increased during the treatment and significant weight loss was not observed in any of the six groups that implying the safety of these preparations. After taking out the tumor at the end of the experiment, we found that the implants were not completely degraded (Figure 7(B–D)). After the extraction of TC-E-5003 from the tumor, HPLC detection showed that the TC-E-5003 group mice had a small amount of ofTC-E-5003 residues, whereas the TC-E-5003-INEI groups had approximately 40% TC-E-5003 residues (Figure 7(J)).

To sum up, the *in vivo* antitumor study confirmed TC-E-5003 in that a combination of the INEI system achieved better antitumor effect while maintaining safety. Therefore, the INEI system is an effective system to develop the new antitumor drug.

### *In vivo* toxic side effect of INEI-paclitaxel

**Figure 8.**
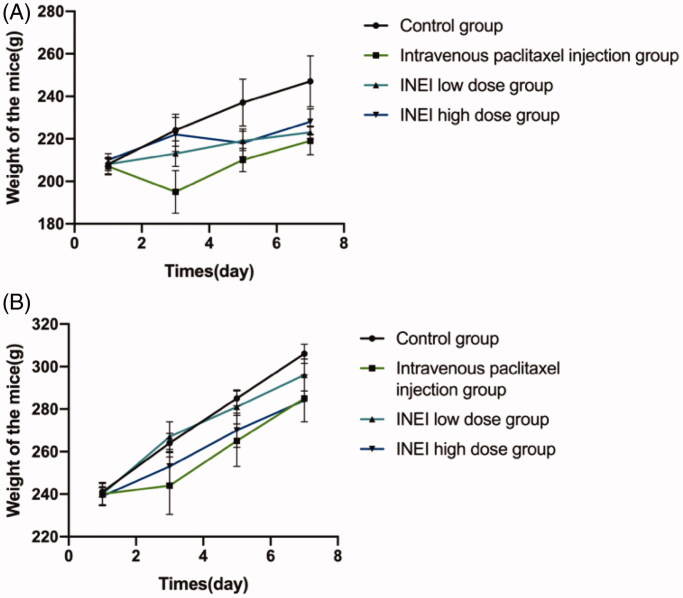
Effects of single administration of different groups on animal weight. (A) Male mice with four group were divided into control group, intravenous paclitaxel injection group, INEI low-dose group and INEI high-dose group (B) Female mice with four group were divided into control group, intravenous paclitaxel injection group, INEI low-dose group and INEI high-dose group.

**Figure 9.**
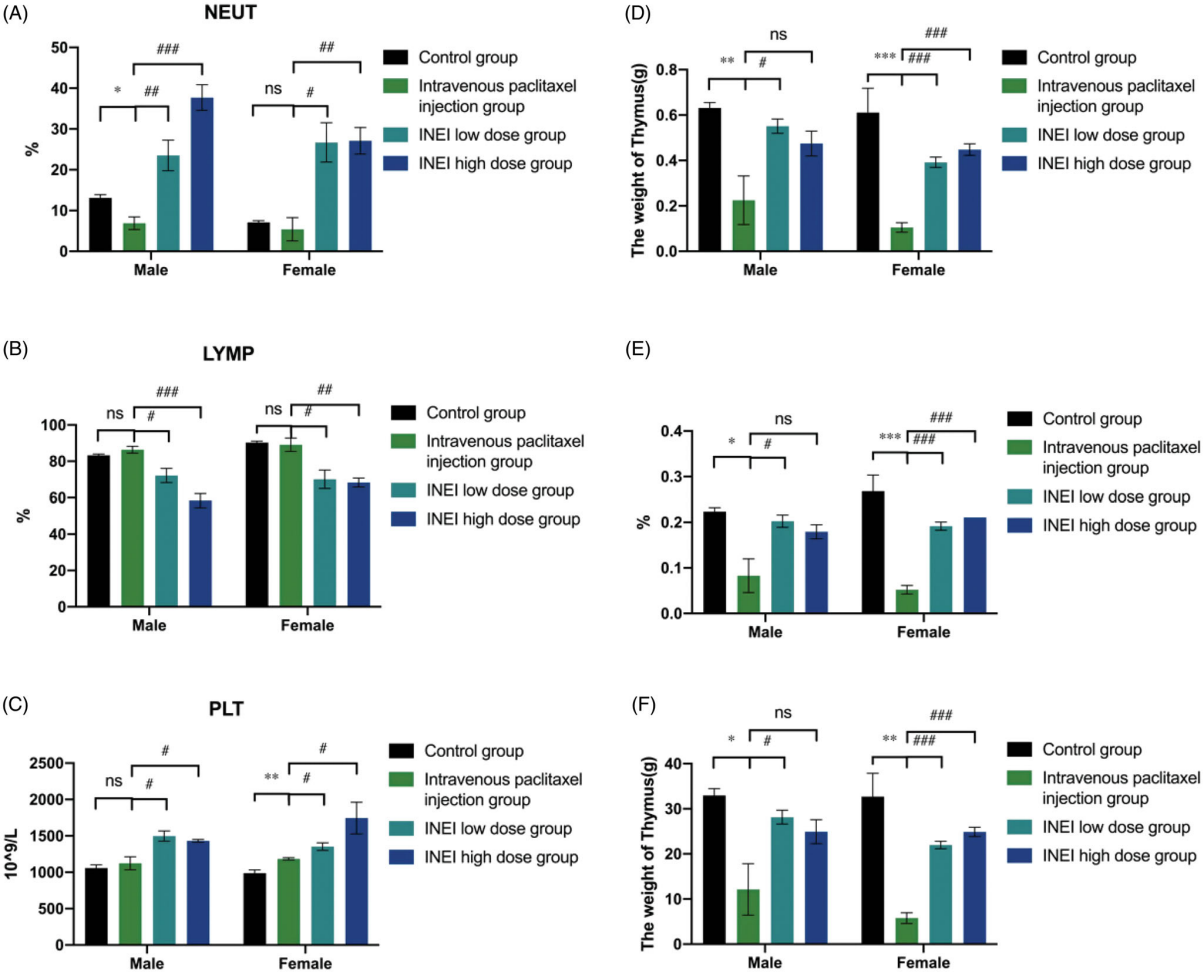
Effects of single administration of different paclitaxel preparations on blood leukocyte classification and organ weight. (A–C) Effects of NEUT% (Neutrophil/White blood cell count), LYMP% (Lymphocyte/White blood cell count) and PLT (Platelet count) on the blood in different paclitaxel preparations. (D–F) Effects of Thymus weight, Thymus relative body mass factor and Thymus relative to brain weight factor in four differ groups. Note: Compared with the control group, *p < .05; **: *p* < .01; ***: *p* < .001; compared with Intravenous paclitaxel injection group, #: *p* < .05; ##: *p* < .01; ###: *p* < .001.

During the observation period, compared with the control group, the weight of the animals in each group increased steadily. Although there was no statistical difference, the weight of the male and female animals in the intravenous paclitaxel group increased from Day3 to Day7 lower than those of the control group and the INEI-low dose group and INEI-High dose group. It is speculated that the weight change of the animals in the intravenous paclitaxel injection group is related to the administration of the paclitaxel injection. The results of weight statistics are shown in Figure 8 and Extended Data Table 3. At the end of the observation period, compared with the control group, the% LYMPH and the% NEUT of the male and female animals in the INEI-low-dose and high-dose groups were significantly increased (*p* < .001). It is speculated that the animals in the sustained-release administration group may have some inflammation. Although the other indicators have statistical differences, the changes are not large, and it is speculated that there is no significant toxicological significance. Blood routine statistics are shown in Figure 9(A–C), Extended Data Table 4–1,2. Compared with the control group, the thymus weight, relative weight, and brain weight of the intravenous paclitaxel injection group were significantly reduced (*p* < .05 ∼ .001). The thymus weight, relative weight, and brain weight of male animals in the INEI-high-dose group were significantly reduced, but The change was smaller than that in the intravenous paclitaxel group. It is speculated that the change of thymus weight in the intravenous paclitaxel injection group is related to the test substance administration. Although the weights of the kidney, adrenal gland, heart, epididymis, and uterus showed significant differences among the groups (*p* < .05), the differences were small or only found in a single sex, without obvious toxicological significance. Statistics of organ weight are shown in Figure 9(D), and Extended Data Table 5–1,2; statistics of organ-body weight coefficient are shown in Figure 9(E) and Extended Data Table 6–1,2; statistics of organ-brain weight coefficient are shown in Figure 9(F) and Extended Data Table 7–1,2.

### H&E assay

**Figure 10.**
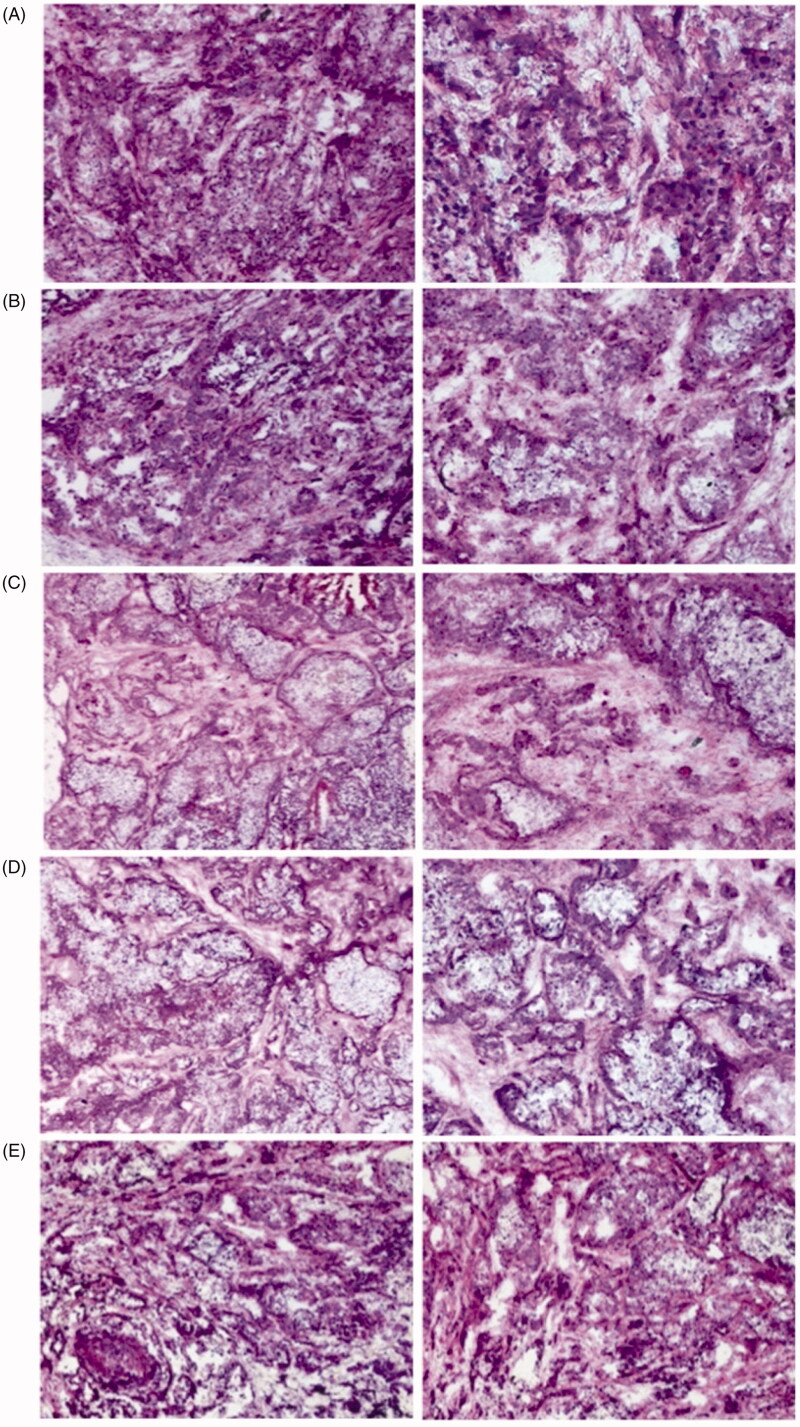
H&E-stained tumor tissues harvested from mice after different treatments. (A) Saline; (B) INEI (40%NBCA); (C) TC-E-5003-40%NBCA; (D) Epirubicin-40%NBCA; and (E) TC-E-5003.

As shown in Figure 10 H&E staining results showed that a large number of normal cancer cells were observed in the saline and 40%NBCA groups. The group treated with TC-E-5003 showed a few necrosis tumor tissues while TC-E-5003-INEI and epirubicin-INEI both showed a few living tumor cells, whereas most of the tumor tissue showed characteristics of necrosis.

## Conclusion

In summary, the INEI drug delivery system is a highly successful site-directed delivery system for tumors in situ. It has the characteristics of fixed-point administration, slow drug release, and the ability to give specific anti-tumor drugs to different tumors. This paper focuses on the application potential of the INEI drug delivery system in the development of new anti-tumor drugs. The experimental results show that the methyltransferase inhibitor TC-E-5003 can be combined with the INEI system to inhibit the growth of tumor cells and induce tumor cell apoptosis. *In vivo* experiments have also shown that the TC-E-5003-INEI system has an excellent tumor-suppressing effect, has a better therapeutic effect than TC-E-5003 alone, and also proves that the system is safe. In summary, the active tumor-targeted delivery system INEI has shown its potential in the development of novel antitumor drugs.

## Supplementary Material

Supplemental Material
