# Supplementary material for: Developing protein arginine methyltransferase 1 (PRMT1) inhibitor TC-E-5003 as an antitumor drug using INEI drug delivery systems
Source: Drug Deliv. 2020 Mar 26;27(1):491–501. doi: 10.1080/10717544.2020.1745327 (PMC7170320; doi:10.1080/10717544.2020.1745327)

Supplementary Materials for

Developing Protein arginine methyltransferase 1 (PRMT1) inhibitor TC-E-5003 as an antitumor drug using INEI drug delivery systems

**Extended Data Table1| Experiment animal group information**

| Sex | Group | Dose（mg/kg） | Number of animals | Animal number |
| --- | --- | --- | --- | --- |
| Male | Control group | 0 | 4 | 1M01-1M04 |
|  | Intravenous paclitaxel injection group | 10 | 4 | 2M01-2M04 |
|  | INEI low dose group | 10 | 4 | 3M01-3M04 |
|  | INEI high dose group | 20 | 4 | 4M01-4M04 |
| Female | Control group | 0 | 4 | 1F01-1F04 |
|  | Intravenous paclitaxel injection group | 10 | 4 | 2F01-2F04 |
|  | INEI low dose group | 10 | 4 | 3F01-3F04 |
|  | INEI high dose group | 20 | 4 | 4F01-4F04 |

**Extended Data Table2| Hematology test index table**

| Indicator name | Abbreviation | Unit | Detection method |
| --- | --- | --- | --- |
| White blood cell count | WBC | 10^9^/L | Peroxidase staining / two-dimensional laser scanning |
| Neutrophil | NEUT | % | Calculation: (NEUT / WBC) * 100% |
| Lymphocyte | LYMP | % | Calculation: (LYMP / WBC) * 100% |
| Monocyte | MONO | % | Calculation: (MONO / WBC) * 100% |
| Eosinophil | EO | % | Calculation: (EOS / WBC) * 100% |
| Basophil | BASO | % | Calculation: (BASO / WBC) * 100% |
| Red blood cell count | RBC | 10^12^/L | Optical laser scattering |
| Hemoglobin | Hb | g/dL | Peroxidase staining |
| Hematocrit | Hct | % | Calculation: (MCV × RBC) / 10 |
| Mean red blood cell volume | MCV | fL | Optical laser scattering |
| Mean hemoglobin content | MCH | Pg | Calculation: HGB / RBC * 103 |
| Mean hemoglobin concentration | MCHC | g/dL | Calculation: HGB / (MCV * RBC) * 106 |
| Platelet count | PLT | 10^9^/L | Optical laser scattering |
| Reticulocyte count | RET | 10^9^/L | Optical laser scattering |

## Extended Data Table3| Effect of single administration of different paclitaxel preparations on animal body weight

| Group | Sex | Weight(g) | | | |
| --- | --- | --- | --- | --- | --- |
|  |  | D1 | D3 | D5 | D7 |
| Control group | ♂ | 241±5 | 264±9 | 285±7 | 306±9 |
| Intravenous paclitaxel injection group | ♂ | 240±10 | 244±27 | 265±24 | 285±22 |
| INEI low dose group | ♂ | 240±11 | 267±14 | 281±16 | 296±15 |
| INEI high dose group | ♂ | 239±8 | 253±16 | 270±16 | 284±15 |
| Control group | ♀ | 208±10 | 224±15 | 237±22 | 247±24 |
| Intravenous paclitaxel injection group | ♀ | 207±7 | 195±20 | 210±11 | 219±13 |
| INEI low dose group | ♀ | 208±6 | 213±12 | 219±9 | 223±6 |
| INEI high dose group | ♀ | 210±6 | 222±16 | 218±13 | 228±12 |

Note: Compared with the control group, all P> 0.05; compared with Intravenous paclitaxel injection group, all P> 0.05.

**
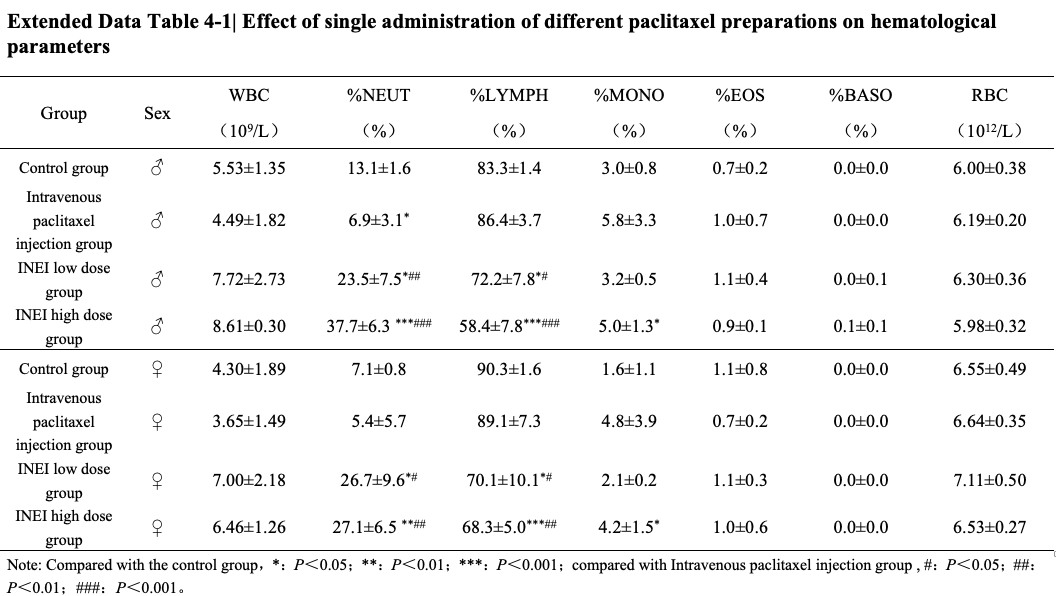
**


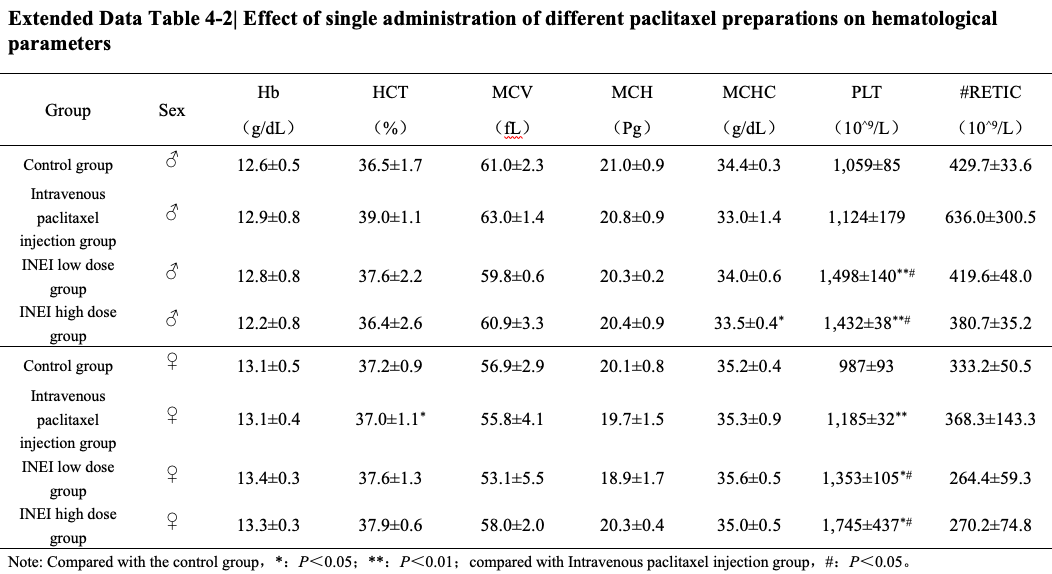


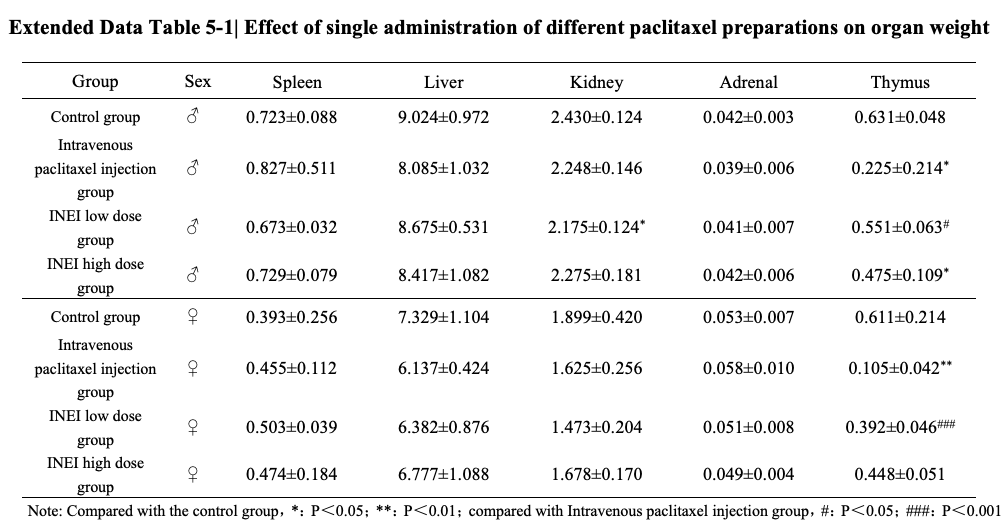


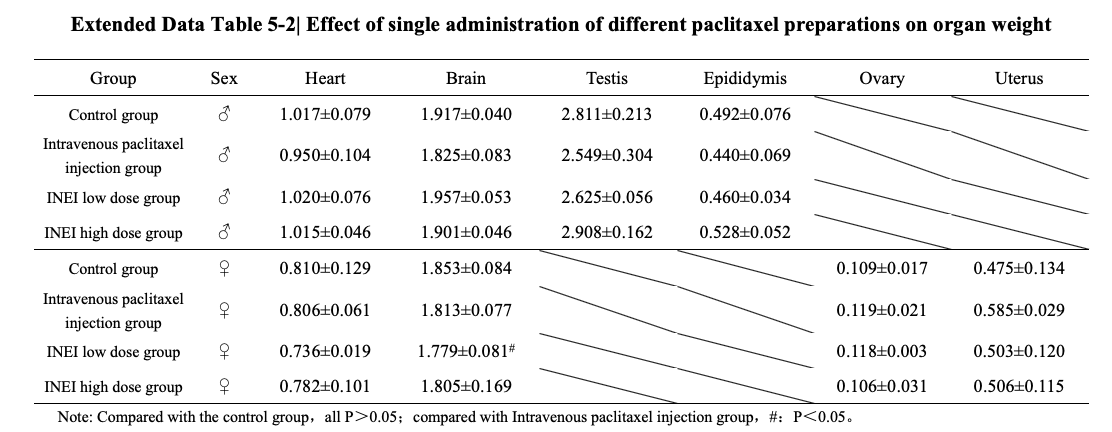


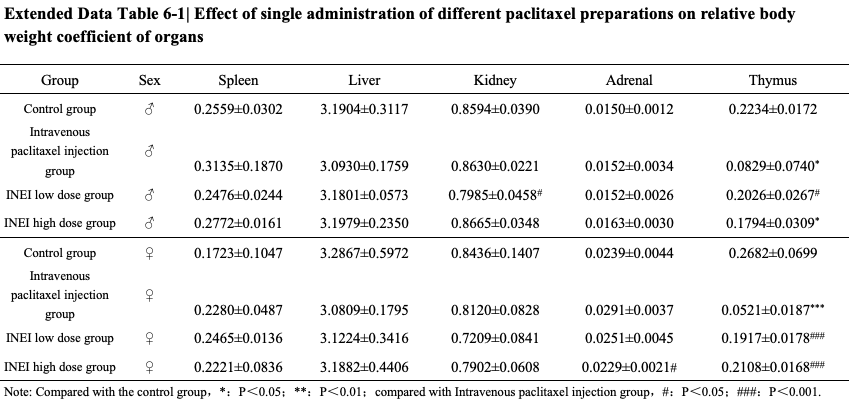


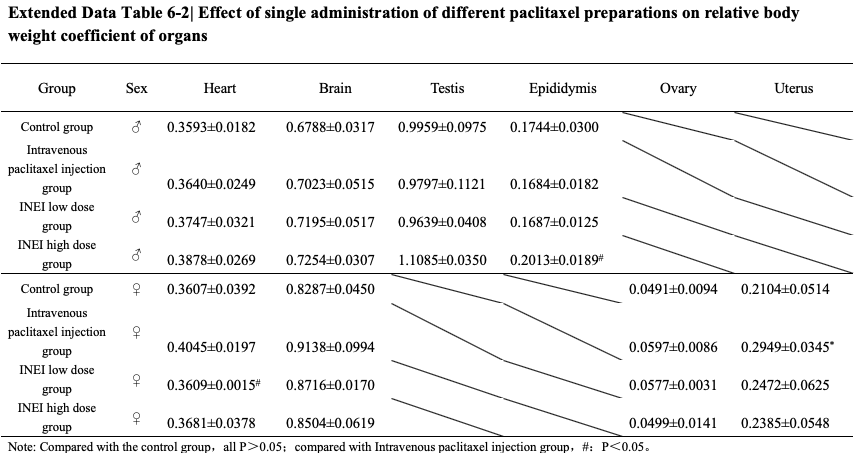


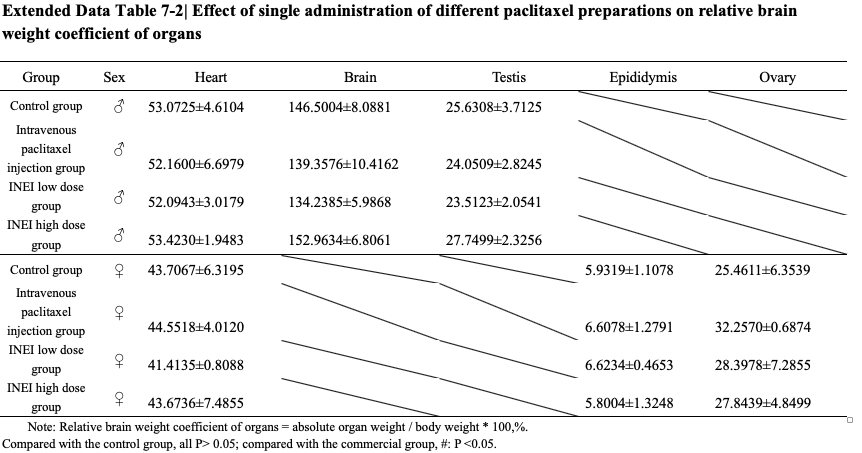

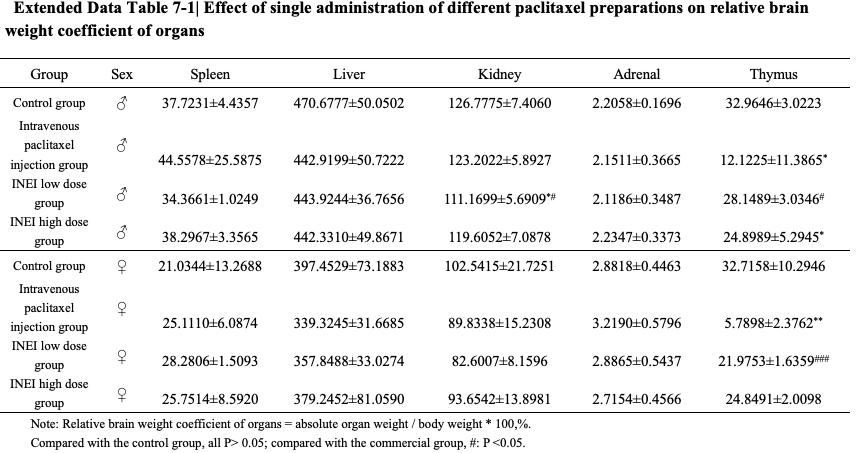

Supplement: Supplemental Material [file IDRD_A_1745327_SM7088.docx]
